# Association between probiotic intervention and sleep quality in the general adult population: a systematic review and meta-analysis

**DOI:** 10.3389/fnut.2026.1795450

**Published:** 2026-03-25

**Authors:** Tingjing Ren, Yilin Wang, Bingnan Zhang, Haiyan Du, Huixia Ren, Naijin Zhang, Hongwu Wang, Yuanyuan Guan

**Affiliations:** 1 School of Public Health, Tianjin University of Traditional Chinese Medicine, Tianjin, China; 2 Institute of Chinese Materia Medica, China Academy of Chinese Medical Sciences, Beijing, China

**Keywords:** adult, meta-analysis, probiotics, sleep quality, systematic review

## Abstract

**Background:**

The discovery of the microbiota–gut–brain axis provides a theoretical basis for using probiotic supplementation to improve sleep health. This meta-analysis systematically evaluated the efficacy of probiotics in adults, including both healthy individuals and those with poor sleep quality.

**Methods:**

Eight public databases were searched to identify relevant randomized controlled trials (RCTs) published before December 2025 to be included in this review. Data from the included studies were extracted, and their risk of bias was assessed. Meta-analysis, sensitivity analysis, and publication bias assessment were conducted using Review Manager 5.4.1.

**Results:**

A total of 13 studies involving 890 participants reported outcomes using the Pittsburgh Sleep Quality Index (PSQI). The meta-analysis revealed that probiotic supplementation significantly reduced PSQI scores compared to the control group (mean difference [MD] = −0.59, 95% confidence interval [CI]: −0.83 to −0.35, *p* < 0.001), indicating moderate heterogeneity (*I*^2^ = 46%). For the Insomnia Severity Index (ISI), the probiotic group also showed significantly lower scores (MD = −0.86, 95% CI: −1.60 to −0.12, *p* = 0.02; *I*^2^ = 26%). In contrast, no significant changes were observed in the Epworth Sleepiness Scale (ESS) scores or serum cortisol levels.

**Conclusion:**

Probiotic supplementation was associated with a modest but statistically significant improvement in sleep quality among adults, regardless of their baseline sleep status (ranging from healthy to suboptimal), as measured by PSQI and ISI scores.

**Systematic review registration:**

https://www.crd.york.ac.uk/PROSPERO/view/CRD420251237016, PROSPERO (CRD420251237016).

## 1 Introduction

Insomnia is a common sleep disorder characterized by difficulty in falling asleep, maintaining sleep, or early awakening, accompanied by symptoms of daytime functional impairment. Globally, the prevalence of insomnia is on the rise. Nearly 40% of adults experience insomnia symptoms, with approximately 10% suffering from chronic insomnia (1). Insomnia not only leads to fatigue and inattention, but in severe cases, can also cause memory decline, emotional disorders, and even neurodegenerative diseases (2). Long-term insomnia significantly increases the risk of chronic diseases, such as cardiovascular diseases, diabetes, and obesity, as well as psychological disorders such as depression and anxiety, imposing a heavy burden on individual’s quality of life and the public health system (3). Poor sleep quality is a characteristic of insomnia. Sleep quality refers to an individual’s self-satisfaction with all aspects of the sleep experience (4) and can be assessed using variables such as sleep efficiency, sleep latency, wake after sleep onset (WASO), and the Pittsburgh Sleep Quality Index (PSQI). Furthermore, sleep quality is influenced by a complex interplay between physiological, psychological, and environmental factors. A recent study connects sleep disorders to reduced quality of life (5). The PSQI has a greater impact on the Satisfaction with Life Scale (SWLS) than the SWLS has on the PSQI (6).

At present, conventional treatment for insomnia primarily includes pharmacological and psychological interventions. While medications such as benzodiazepines demonstrate short-term efficacy, their use is often limited by adverse reactions, including dependency and tolerance (7). Psychological approaches, particularly cognitive behavioral therapy for insomnia (CBT-I), have demonstrated sustained benefits; however, their widespread implementation is hindered by a shortage of trained professionals and high treatment costs (8). Emerging evidence suggests that dietary patterns may also influence sleep. Specifically, plant-based diets rich in whole grains, dairy products, and lean proteins, including fish, appear to confer the greatest benefits for sleep health (9).

Research into the “microbiota-gut-brain axis” has shown that the gut microbiota play an important role in sleep regulation. The gut microbiota communicates bidirectionally with the central nervous system through neuroendocrine, immune, and metabolic pathways, thereby influencing the sleep–wake cycle (10, 11). A recent study indicates that butyrate, a metabolite produced by gut microbes, promotes sleep by regulating neuronal activity in the lateral hypothalamic area in mice. In insomnia patients, lower serum butyrate levels and a deficiency of butyrate-producing species in the gut microbiota were observed (12). These findings provide a theoretical basis for improving sleep by targeting and regulating the gut microbiota. Given the role of the gut microbiota in sleep regulation, probiotics have emerged as a potential treatment for poor sleep quality. Therefore, this systematic review and meta-analysis aims to assess the impact of consuming probiotics on sleep quality.

## 2 Methods

The protocol was registered in PROSPERO (CRD420251237016), and this study was conducted in accordance with the Preferred Reporting Items for Systematic Reviews and Meta-analysis (PRISMA) (13) guidelines.

### 2.1 Literature retrieval strategy

We conducted a search in PubMed, Embase, Cochrane Library, Web of Science, China National Knowledge Infrastructure (CNKI), Wanfang, China Science and Technology Journal Database (VIP), and Sinomed databases. The search terms were probiotic* AND (“sleep quality” OR “sleep disorder” OR insomnia) AND “random controlled trial”. The search spanned from database inception to 31 December 2025, with no language or other restrictions. Studies were screened by one reviewer and independently checked by a second reviewer. Any discrepancies were resolved through discussion or third-party arbitration.

### 2.2 Eligibility criteria

The inclusion criteria for this systematic review were as follows: I. A randomized controlled trial (RCT) design assessing the effects of probiotics on sleep quality; II. Probiotics as the primary intervention compared to a non-intervention control; III. Study population consisting of adults, and IV. Report of at least one relevant sleep outcome. The most consistently reported measure was the PSQI. The secondary outcomes, reported by a subset of studies, included the Insomnia Severity Index (ISI), the Epworth Sleepiness Scale (ESS), and changes in serum cortisol levels. All included studies provided the mean, standard deviation, and participant count for each group at both baseline and postintervention.

The exclusion criteria were as follows: I. Duplicate publications; II. Studies available only as abstracts; and III studies with incomplete or inaccessible data.

### 2.3 Data extraction

Data were extracted by one reviewer and independently checked by a second reviewer. The following information was collected: participant characteristics, intervention details, and outcome data. Any discrepancies were resolved through discussion or third-party arbitration.

The primary outcome was sleep quality, assessed using the PSQI. The PSQI consists of 19 items that generate 7 component scores, which are summed to yield a global score ranging from 0 to 21. Higher scores indicate poorer sleep quality. Typically, a PSQI global score >7 is considered to indicate poor sleep quality. Secondary outcomes included patient-reported outcomes, specifically the ISI for insomnia severity, and the ESS for daytime sleepiness, as well as serum cortisol levels. Serum cortisol serves as a biomarker for the body’s stress response system. Elevated nighttime cortisol is a well-established neuroendocrine correlate of the hyperarousal that contributes to insomnia, linking subjective sleep complaints to objective physiological dysregulation. We also extracted additional objective sleep parameters, including non-rapid eye movement stage 3 (N3), rapid eye movement (REM), and non-rapid eye movement (NREM) sleep stages, as measured by wearable devices or polysomnography (PSG) that incorporated electroencephalography (EEG).

### 2.4 Risk of bias assessment

The quality of RCTs was evaluated using the Cochrane Risk of Bias Tool (RoB2.0). Assessments were performed across five domains, namely randomization process, deviations from intended interventions, missing outcome data, measurement of the outcome, and selection of the reported result. Following the standardized algorithm, each domain was categorized as “low risk of bias,” “some concerns,” or “high risk of bias.” The overall risk was determined as “low risk of bias” if all domains were deemed low risk. It was categorized as “some concerns” if at least one domain showed some concerns but no domain was rated as high risk. The overall risk was determined as “high risk of bias” if at least one domain was judged to be high risk. In cases of missing information, authors were contacted to provide the unavailable data from their published reports.

### 2.5 Statistical analysis

Meta-analysis was conducted using Review Manager 5.4.1. Continuous variables were analyzed using the mean difference (MD) and a 95% confidence interval (CI). A fixed-effects model was applied if heterogeneity was low (*I*^2^ < 50%), and a random-effects model was used if heterogeneity was high (*I*^2^ > 50%). If moderate heterogeneity was observed, results from both fixed-effect and random-effects models were compared to assess whether the pooled estimates were disproportionately influenced by the study weight distribution or by the presence of heterogeneity. Consistency between the two models would strengthen confidence in the findings.

Change-from-baseline scores were used as an outcome, and the standard deviations (SDs) were imputed using a correlation coefficient (*r*) of 0.7, based on the baseline SDs and follow-up SDs, according to the formula recommended by the Cochrane Handbook:


$$SD_{\text{change}}=\sqrt{SD_{\mathrm{pre}}^{2}+SD_{\text{post}}^{2}-(2r\times SD_{\mathrm{pre}}\times SD_{\text{post}})}$$

A *p*-value of < 0.05 was considered statistically significant for the overall effect estimates and subgroup analyses, whereas for assessing heterogeneity, a *p*-value of < 0.10 was considered statistically significant. Subgroup analyses were conducted based on factors such as participants’ source, mean age, baseline insomnia severity, and intervention duration. Sensitivity analysis was performed to evaluate the robustness of the results. If the size of the combined effect remains stable after excluding each study in sequence, the result is considered robust. The funnel plot was used to evaluate publication bias, and the symmetrical scatter distribution indicated no bias.

### 2.6 Rating quality of evidence

We rated the quality of evidence for each outcome using the Grading of Recommendations, Assessment, Development and Evaluation (GRADE) approach. Since the RCTs are categorized as the highest level of evidence, they were evaluated for potential downgrading based on five factors: limitations, inconsistency, indirectness, inaccuracy, and publication bias. The certainty of the evidence was then categorized as high, moderate, low, or very low.

## 3 Results

### 3.1 Included studies

**Figure 1:**
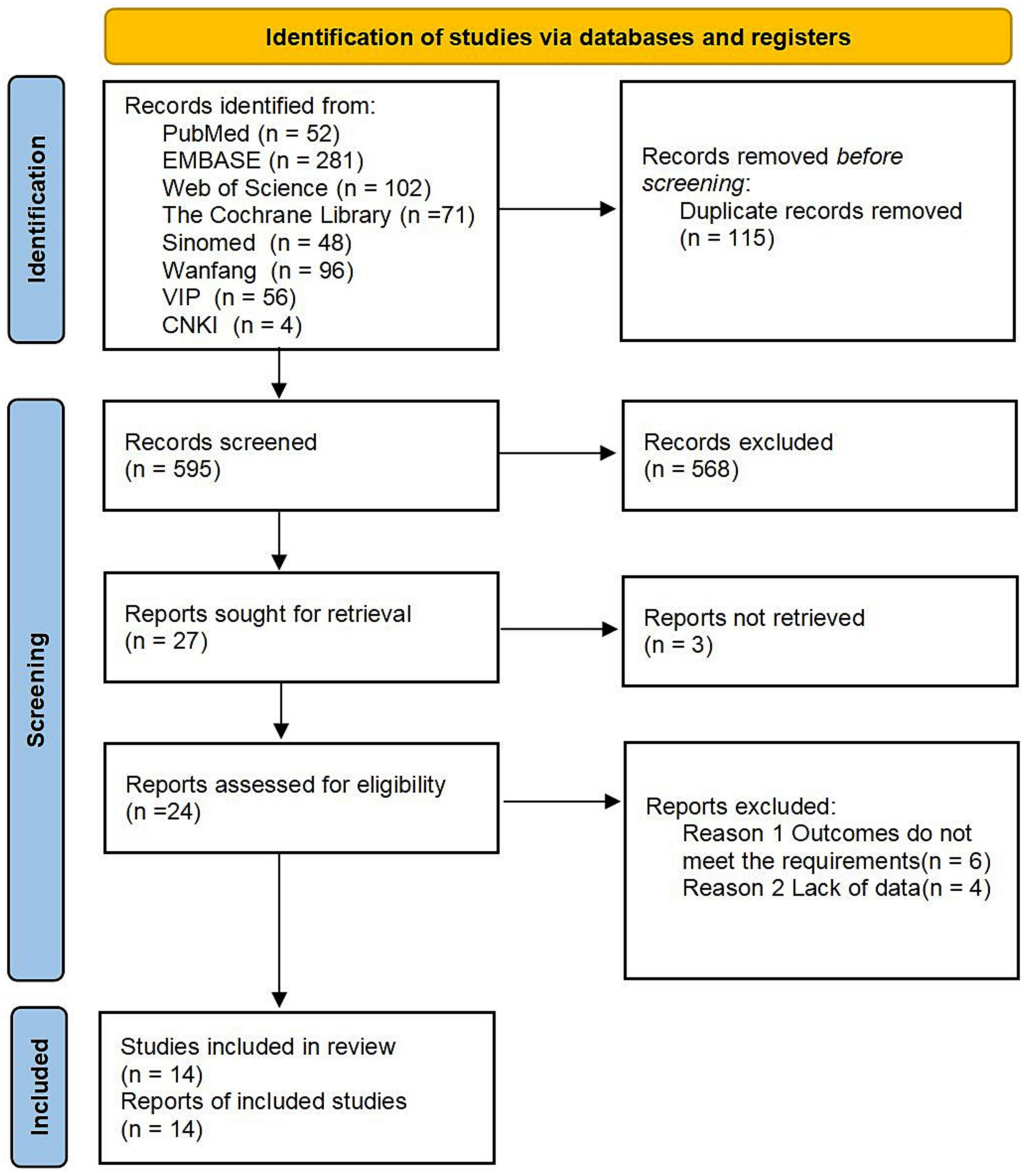
Study selection procedure according to the PRISMA statement.

A total of 710 relevant studies were identified through the database search: 52 from PubMed, 281 from Embase, 102 from Web of Science, 71 from the Cochrane Library, 4 from CNKI, 96 from Wanfang, 51 from VIP, and 48 from SinoMed. During screening, 115 studies were excluded due to duplication, and 568 were excluded for failing to meet the inclusion criteria. A total of 14 studies (14–27) were ultimately included, as shown in Figure 1.

The studies included were recent, ranging from 2019 to 2025. The total sample size across all studies was 946 participants. Details of the study characteristics are presented in Table 1.

**Table 1:** Study characteristics.

Author	Nation/district	Sample size (T/C)	PSQI baseline (T/C)	ISI baseline (T/C)	Mean age (T/C)	Male ratio	Treatment	Total CFU/day	Treatment weeks	Comparison	Outcome measured
Boehme et al. 2023 (14)	Swiss	24/21	4.80 ± 2.20/5.20 ± 1.80		37.50 ± 10.00/40.70 ± 9.00	58%	*Bifidobacterium longum* NCC3001	1.0 × 10^10^	6	Placebo	PSQI
Grant et al. 2025 (15)	USA	25/31		10.24 ± 3.67/13.11 ± 4.27	39.00 ± 13.00	37%	*Lactiplantibacillus plantarum* Lp815	5.0 × 10^9^	6	Placebo	ISI
Ho et al. 2021 (16)	Taiwan	21/19	12.33 ± 2.20/11.26 ± 2.33	17.52 ± 3.49/16.74 ± 3.03	26.43 ± 5.95/25.47 ± 4.64	33%	*Lactobacillus plantarum* PS128	3.0 × 10^10^	4	Placebo	PSQI, ISI, ESS
Kerksick et al. 2024 (17)	USA	35/35	5.43 ± 2.40/5.41 ± 2.90		29.70 ± 9.00/32.30 ± 10.00	50%	*Limosilactobacillus fermentum* LF16, *Lacticaseibacillus rhamnosus* LR06, *Lactiplantibacillus plantarum* LP01, *Bifidobacterium longum*04	4.0 × 10^9^	4	Placebo	PSQI
Lan et al. 2023 (18)	China	20/20	11.60 ± 3.17/10.10 ± 2.29		38.95 ± 10.59/36.55 ± 11.31	35%	*Bifidobacterium breve* CCFM1025	1.0 × 10^9^	4	Placebo	PSQI, COR
Lee et al. 2021 (19)	Korea	63/59	8.16 ± 3.04/7.22 ± 2.29	11.46 ± 4.54/10.03 ± 4.84	38.86 ± 10.89/37.63 ± 11.04	32%	*Lactobacillus reuteri* NK33, *Bifidobacterium adolescentis* NK98	2.5 × 10^9^	8	Placebo	PSQI, ISI, COR
Li et al. 2024 (20)	China	37/34	5.73 ± 2.81/4.47 ± 2.04		23.56 ± 2.33/23.58 ± 2.10	24%	*Bifidobacterium breve* 207–1	5.0 × 10^10^	4	Placebo	PSQI, COR
Li et al. 2024 b (21)	China	37/34	5.85 ± 2.74/4.97 ± 2.48		25.17 ± 2.78/25.02 ± 4.32	25%	*Lacticaseibacillus paracasei* 207–27	1.0 × 10^10^	4	Placebo	PSQI
Liu et al. 2025 (22)	China	53/53	11.40 ± 2.60/11.50 ± 2.60	13.90 ± 3.40/14.40 ± 3.40	19.90 ± 1.30/19.90 ± 1.40	38%	*Bifidobacterium animalis* subsp. *lactis* BLa80	1.0 × 10^10^	8	Placebo	PSQI, ISI
Marotta et al. 2019 (23)	Italy	18/15	5.61 ± 2.17/4.67 ± 2.61		21.61 ± 2.20/21.67 ± 2.19	64%	*Limosilactobacillus fermentum* LF16, *Lacticaseibacillus rhamnosus* LR06, *Lactiplantibacillus plantarum* LP01, *Bifidobacterium longum*04	4.0 × 10^9^	6	Placebo	PSQI
Murakami et al. 2024 (24)	Japan	61/65	7.60 ± 1.60/7.60 ± 1.50		46.10 ± 7.00/46.70 ± 7.30	49%	*Bifidobacterium adolescentis* SBT2786	more than 1.0 × 10^11^	4	Placebo	PSQI, ESS
Nishida et al. 2019 (25)	Japan	29/31	4.20 ± 2.15/3.40 ± 2.23		24.90 ± 2.69/25.30 ± 3.34	68%	*Lactobacillus gasseri* CP2305	1.0 × 10^10^	24	Placebo	PSQI
Nobile et al. 2023 (26)	Italy	12/12	5.50 ± 1.94		36.00 ± 9.00	38%	*Limosilactobacillus reuteri* PBS072, *Bifidobacterium breve* BB077	4.0 × 10^9^	4	Placebo	PSQI
Patterson et al. 2024 (27)	Ireland	43/44	5.70 ± 2.20/5.80 ± 2.80		32.43 ± 7.91/30.13 ± 8.41	28%	*Bifidobacterium longum* 1714	1.0 × 10^9^	8	Placebo	PSQI, ESS

T, treatment group; C, control group; PSQI, Pittsburgh Sleep Quality Index; ISI, Insomnia Severity Index; ESS, Epworth sleepiness scale; COR, cortisol.

### 3.2 Methodological quality: assessment of bias

**Figure 2:**
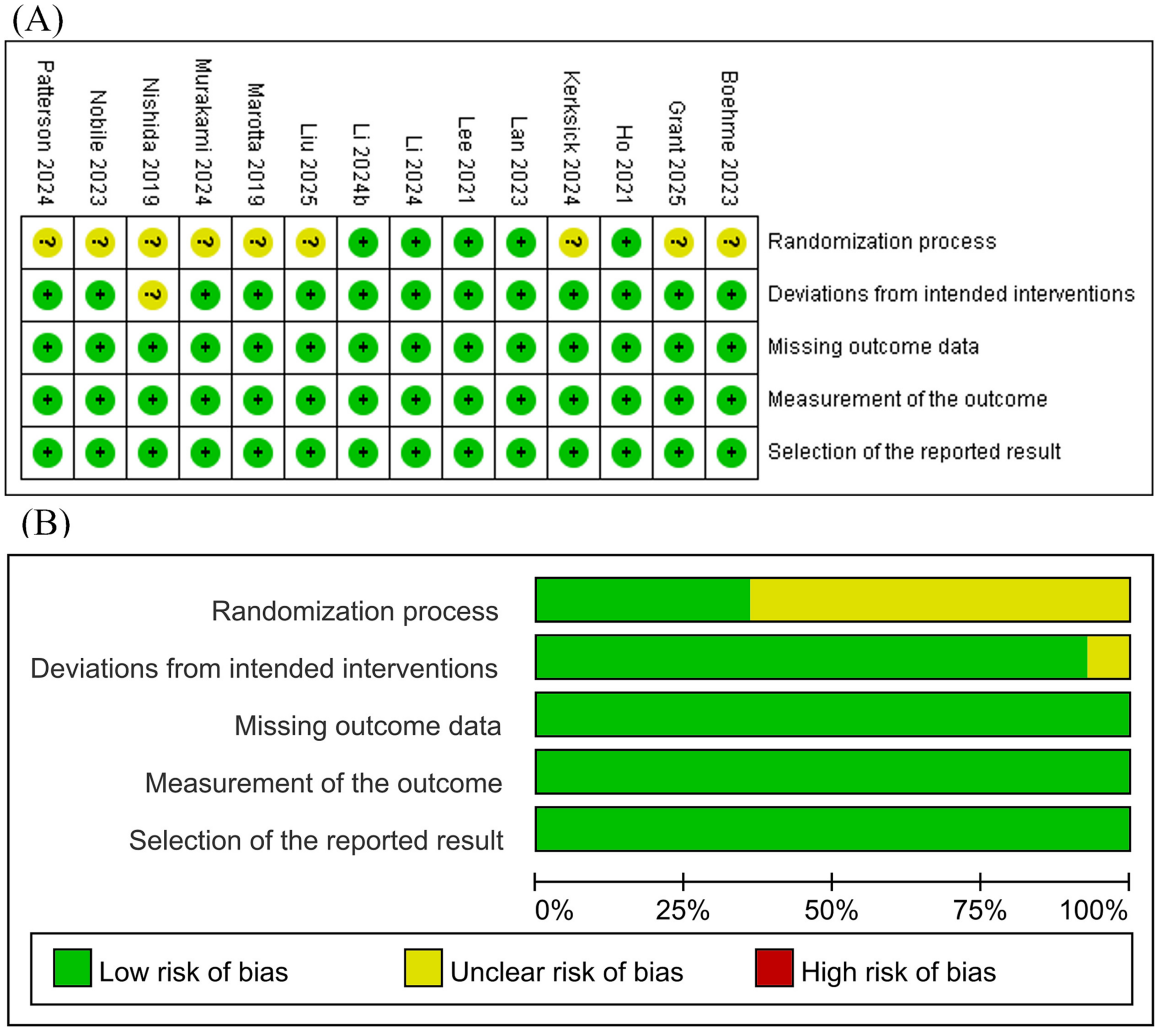
Risk of bias assessment. **(A)** Risk of bias summary and **(B)** risk of bias graph.

The Cochrane Risk of Bias tool (Rob2.0) was used to evaluate the included studies. Nine studies had an unclear risk of bias in the randomization process due to insufficient reporting of randomization methods. One study had an unclear risk of bias due to the research environment. All other domains were assessed as low risk. Overall, five studies were rated as low risk of bias, and nine studies raised concerns, as shown in Figure 2.

### 3.3 Meta-analysis results

#### 3.3.1 Primary outcome

**Figure 3:**
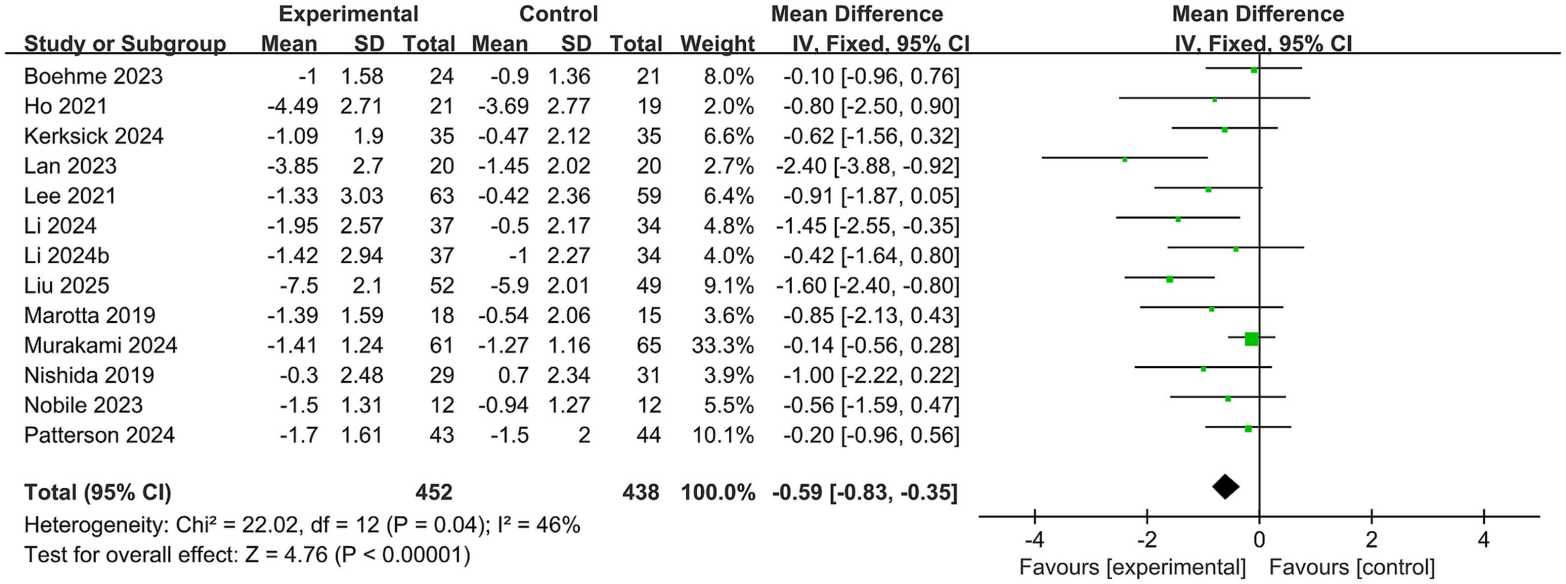
Forest plot for the effect of probiotics on PSQI scores.

The PSQI was reported in 13 trials (14, 16–27), involving a total of 890 participants. A fixed-effect model was used to estimate the effect size because of low heterogeneity (*I*^2^ < 50%). PSQI scores were significantly reduced in the probiotic group compared to the control group (MD −0.59, 95%CI −0.83 to −0.35, *I*^2^ = 46%, *p* < 0.001), as shown in Figure 3. There was clear conceptual heterogeneity across the studies, specifically regarding strains, dosages, and intervention durations. To assess the robustness of our primary findings, we performed a sensitivity analysis using a random-effects model (MD −0.74, 95% CI −1.10 to −0.38) (see Supplementary Figure S10). The consistency between the two models suggests that the overall finding is robust and not substantially influenced by the choice of statistical model, despite moderate heterogeneity.

Subgroup analysis was conducted to assess heterogeneity in PSQI scores, as shown in Table 2. Studies were stratified by source of participants (Asia, Europe, and America), intervention duration (4 weeks vs. >4 weeks), and baseline insomnia severity (PSQI scores ≥7 or <7). An additional analysis was performed by grouping studies by the probiotic genus used (*Bifidobacterium*, *Lactobacillus*, or both) and by sample size, with studies categorized as small (<30 participants) or large (≥30 participants). None of these subgroup analyses revealed statistically significant differences (see Supplementary Figures S1–6).

**Table 2:** Subgroup analyses of the primary efficacy outcome—PSQI.

Subject	Subgroup	Number of studies	*I* ^2^	MD (95%CI)	*p*-value
Source of participants	Asia	8	63%	−0.69 (−0.99, −0.39)	<0.00001
	Europe and America	5	0%	−0.39 (−0.80, 0.03)	0.07
Intervention duration	4 weeks	7	51%	−0.49 (−0.80, −0.17)	0.003
	>4 weeks	6	43%	−0.74 (−1.11, −0.36)	0.0001
Baseline insomnia severity	PSQI ≥ 7	5	76%	−0.62 (−0.95, −0.29)	0.0002
	PSQI < 7	8	0%	−0.55 (−0.91, −0.20)	0.002
Mean age	≥30 years old	7	43%	−0.37 (−0.66, −0.09)	0.01
	<30 years old	6	0%	−1.16 (−1.62, −0.70)	<0.00001
Genus	*Bifidobacterium*	6	74%	−0.61 (−0.93, −0.28)	0.0003
	*Lactobacillus*	3	0%	−0.73 (−1.50, −0.04)	0.06
	*Bifidobacterium* and *Lactobacillus*	4	0%	−0.73 (−1.24, −0.21)	0.006
Sample size	Small	5	9%	−0.42 (−0.72, −0.12)	0.006
	Large	8	22%	−0.34 (−0.49, −0.19)	<0.00001

**Figure 4:**
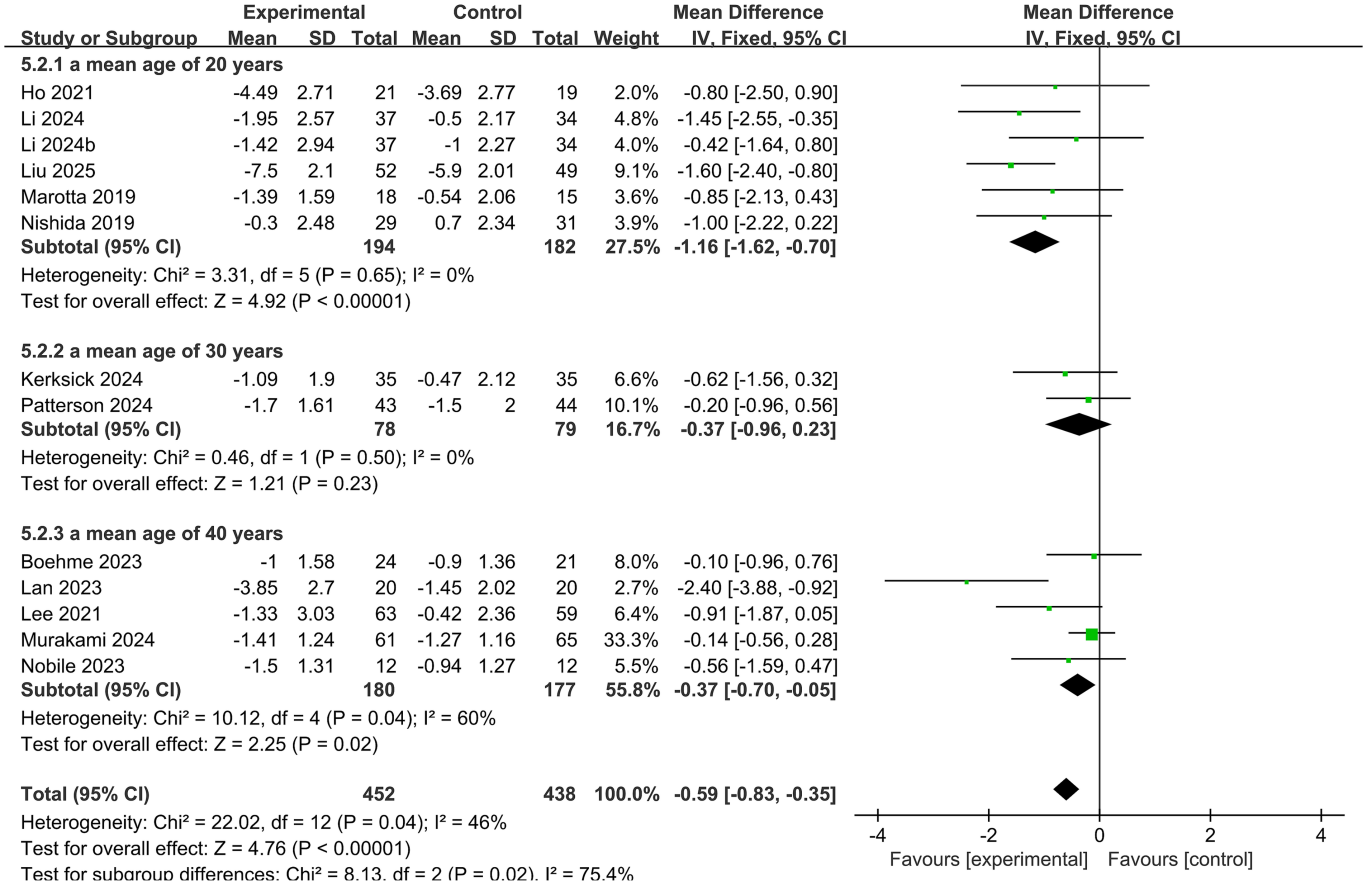
Forest plot for the effect of probiotics on PSQI scores, stratified by mean participant age.

To examine the potential moderating effect of age, studies were stratified by the mean age of participants; the results are presented in Figure 4. The subgroup with a younger mean age (<30 years) showed a better treatment effect than the older subgroup (≥30 years) (test for subgroup differences: *p* = 0.02, *I*^2^ = 75.4%).

#### 3.3.2 Secondary outcomes

**Figure 5:**
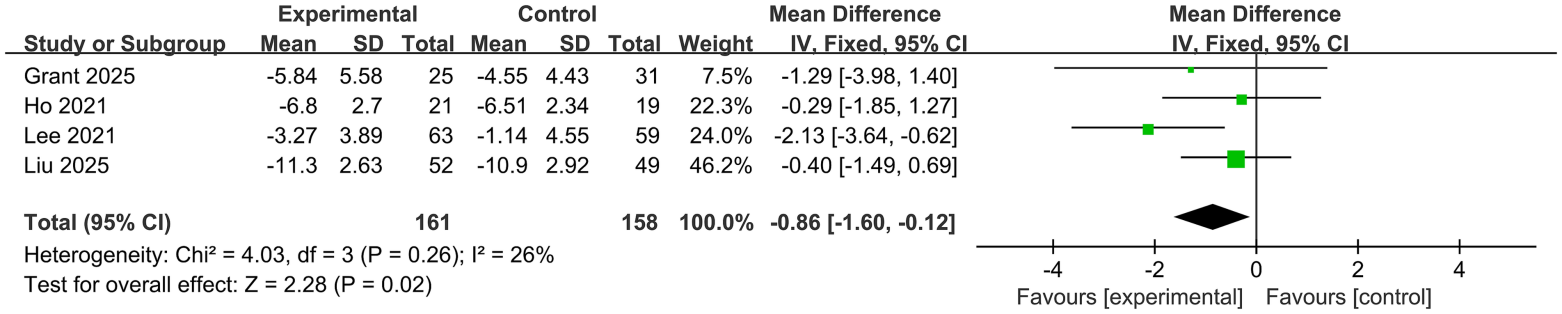
Forest plot for the effect of probiotics on ISI scores.

ISI was reported in 4 trials (15, 16, 19, 22), with a total of 319 participants. ISI scores were significantly reduced in the probiotic group compared to the control group (MD −0.86, 95%CI −1.60 to −0.12, *p* = 0.02), as shown in Figure 5. The heterogeneity was low (*I*^2^ = 26%).

**Figure 6:**
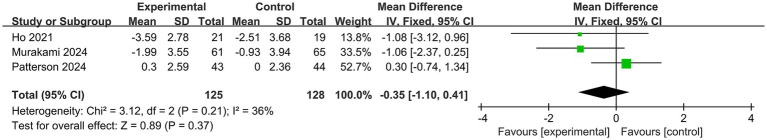
Forest plot for the effect of probiotics on ESS scores.

ESS scores, reported in three trials involving 253 participants (16, 24, 27), showed no significant change (MD −0.35, 95% CI −1.10 to 0.41, *p* = 0.37). There was low heterogeneity among the included studies (*I*^2^ = 36%). These results are presented in Figure 6.

**Figure 7:**
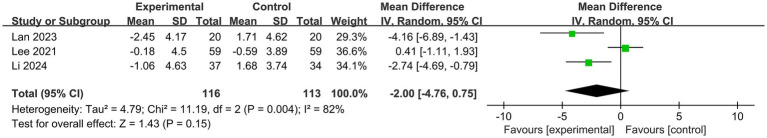
Forest plot for the effect of probiotics on serum cortisol levels.

As shown in Figure 7, the pooled estimate from three trials (*n* = 229) (18–20) indicated a non-significant trend toward lower serum cortisol levels in the intervention group (MD −2.00 μg/dL, 95% CI −4.76 to 0.75 μg/dL, *p* = 0.15), with high heterogeneity (*I*^2^ = 82%).

### 3.4 Narrative synthesis of objective sleep outcomes

Four of the included trials reported objective sleep measures, as shown in Table 3. Due to the limited number of studies and differences in outcome measures, a formal meta-analysis was not possible. Two studies (21, 24) reported that the intervention groups showed a significantly longer sleep duration than the placebo groups. Nishida et al. (25) reported no significant changes in total REM/NREM sleep time, but found that CP2305 significantly increased the EEG δ-power ratio in the first sleep cycle and significantly shortened N3 latency and WASO compared to placebo. Ho et al. (16) reported that the PS128 group had significantly fewer awakenings during N3 sleep than placebo on Day 30. While overall EEG power spectra showed no significant differences, the PS128 group demonstrated higher *δ* power percentage and lower *β*, *α*, and *θ* power percentages than controls.

**Table 3:** Summary of findings on objective sleep outcomes.

Study	Intervention	Sleep duration & continuity	Sleep architecture	Sleep depth (EEG power)
Li et al. b (21)	207–27	↑ Total sleep time (+1.04 h vs. placebo)	—	—
Murakami et al. (24)	SBT2786	↑ Total sleep time ↑ Sleep/↓ Wake (last 2 h of sleep)	↑ REM sleep time	—
Nishida et al. (25)	CP2305	↓ WASO	No Δ in REM/NREM time	↓ N3 latency ↑ δ power ratio (1st sleep cycle)
Ho et al. (16)	PS128	↓ N3 awakenings (day 30)	—	↑ δ power % ↓ β%, α%, θ%

↑: significant increase, ↓: significant decrease, No Δ: no significant change, —: not reported, WASO: wake after sleep onset, N3: non-rapid eye movement sleep stage 3, REM: rapid eye movement sleep, NREM: non-rapid eye movement sleep.

### 3.5 Sensitivity analysis

To assess the robustness of the primary outcome, a sensitivity analysis was performed using the leave-one-out method. The results demonstrated that the pooled MD of PSQI and its statistical significance remained stable when each study was omitted in turn. This indicates that our meta-analysis conclusions are not driven by any single influential study.

### 3.6 Publication bias

**Figure 8:**
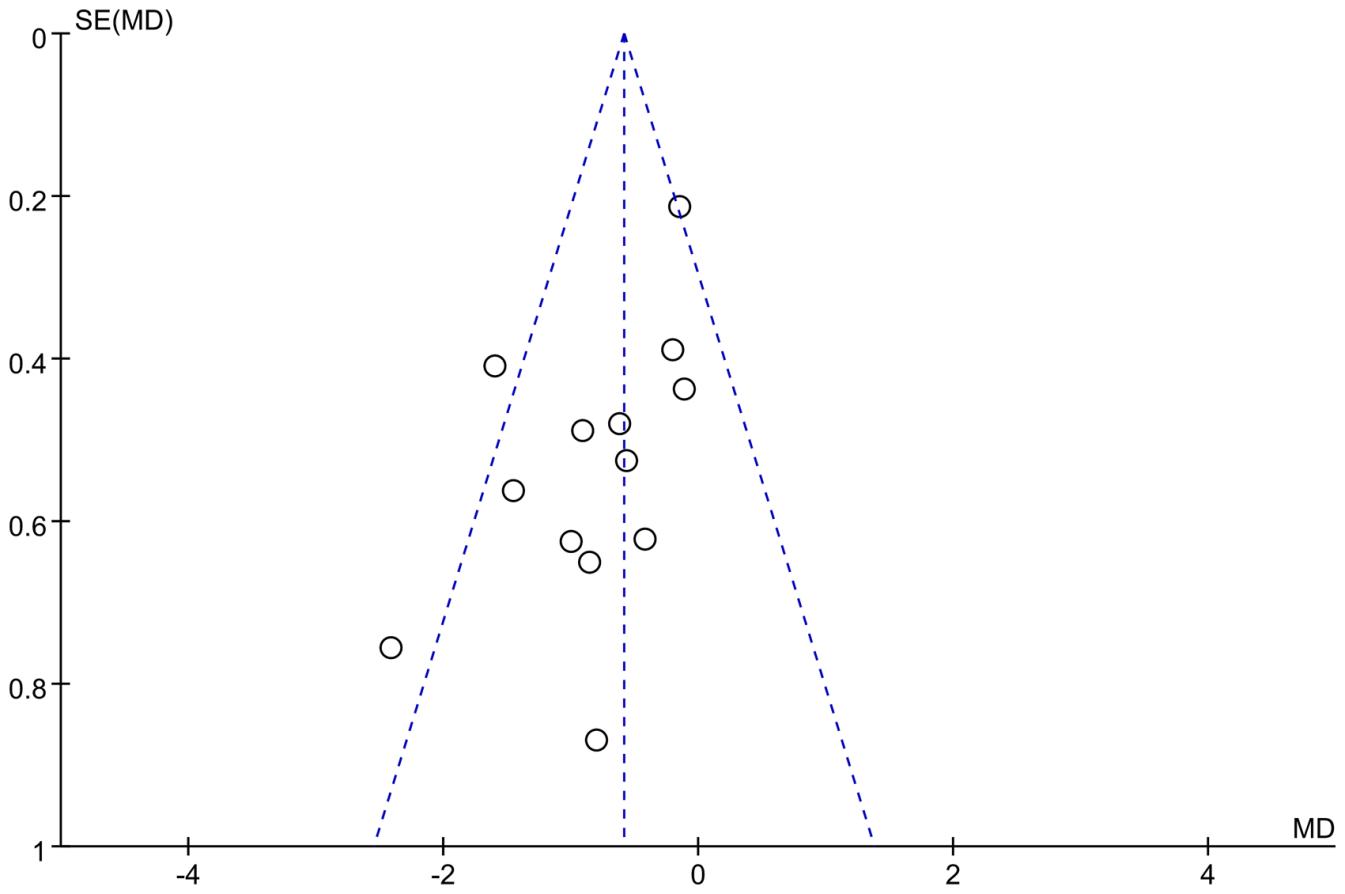
Funnel plot for PSQI outcomes.

The results of funnel plots for PSQI showed symmetrical scatter distributions, as shown in Figure 8. Begg’s rank correlation test did not indicate statistically significant publication bias (*z* = −1.46, *p* = 0.143). The *p*-value of Egger’s test was 0.0868 (>0.05), but it was close to the critical level. Furthermore, the bias estimate was negative, indicating a potential risk of publication bias that might lead to an overestimation of the effect size.

### 3.7 Quality of evidence

It is considered that RCTs are set at the highest level of evidence in the evaluation of GRADE evidence quality. Thus, only five degradation factors (risk of bias, inconsistency, indirectness, imprecision, and publication bias) are considered. The results are shown in Table 4. The evidence for serum cortisol was downgraded by one level for inconsistency (*I*^2^ > 50%) and one level for publication bias based on funnel plot analysis (see Supplementary Figures S7–9). Additionally, ISI, ESS, and serum cortisol were downgraded for imprecision because their respective sample sizes (319, 253, and 229) fell below the threshold of 400 according to the GRADE Working Group (28). Overall, the evidence quality was rated high for PSQI, moderate for ISI and ESS, and very low for cortisol.

**Table 4:** Quality rating of evidence using the GRADE approach.

Outcome	Risk of bias	Inconsistency	Indirectness	Imprecision	Publication bias	Quality of evidence
PSQI	None	None	None	None	None	⊕ ⊕ ⊕ ⊕ High
ISI	None	None	None	Serious	None	⊕ ⊕ ⊕ ○ Moderate
ESS	None	None	None	Serious	None	⊕ ⊕ ⊕ ○ Moderate
COR	None	Serious	None	Serious	Serious	⊕ ○ ○ ○ Very low

## 4 Discussion

### 4.1 Main findings

The review synthesizes evidence from 14 studies conducted across eight countries, involving a total of 946 participants, with the primary aim of systematically evaluating the association between probiotic supplementation and sleep quality or insomnia. This systematic review incorporates seven latest studies published in 2024 and 2025, representing an updated synthesis compared to existing systematic reviews on the same topic. Although serum cortisol concentration was considered a secondary outcome measure due to its objectivity, no statistically significant differences were observed between the groups.

PSQI was selected as the primary efficacy outcome given its widespread use and reliability in assessing sleep quality (29). Given the relatively small heterogeneity identified in the meta-analysis, the conclusions drawn from these independent studies are convergent and the evidence is relatively stable. It enhances the credibility of our merger results. PSQI scores were significantly reduced in the probiotic group than in the control group in our study. This is consistent with prior meta-analyses (30–32). Irwin et al. (30) reported that, using a random-effects model, probiotic supplementation significantly reduced the PSQI score relative to baseline (MD: −0.78, 95% CI: −1.166 to −0.395; *p* < 0.001). According to Ito et al. (31), the random-effects model demonstrated a statistically significant reduction in PSQI scores within the probiotics group at 4–6 weeks (MD: −0.56; 95% CI: −0.97 to −0.22) and at 8–16 weeks (MD: −0.53; 95% CI: −0.92 to −0.14). Yu et al. (32) used a random-effects model in analyzing the 11 included studies, yielding an overall effect size of −0.34 (95% CI: −0.56 to −0.13), with moderate heterogeneity (*I*^2^ = 42.6%, *p* = 0.001). Collectively, these findings indicate that probiotic supplementation improves sleep quality. Another study (33) reported a bigger effect size of −2.10 but included only six articles, two of which lacked placebo controls, thereby limiting the strength of its conclusions. Overall, current evidence suggests that the average effect size of probiotics on reducing total PSQI scores is close to 0.59, and did not reach the minimum clinically important difference (MCID of 1.5–3.0), thus the clinical significance is limited. The magnitude of improvement is smaller than that achieved through pharmacological treatments, CBT (34), acupuncture (35), or exercise (36). Probiotics represent a viable non-pharmacological intervention that may be used as an adjunctive therapy to enhance sleep outcomes.

### 4.2 Subgroup analyses

The probiotics examined in this study included strains from the genera *Bifidobacterium* and *Lactobacillus*, either administered individually or in combination. Both genera are widely recognized as common beneficial probiotics and have been shown to exert multiple physiological regulatory functions (37). Subgroup analysis based on genus showed no statistically significant differences. However, a growing body of evidence indicates that probiotic effects are often strain-specific (38). Different strains may have distinct biological effects, even within the same genus, so we should not overgeneralize the findings to all probiotics in a single group.

Subgroup analysis by participant age category revealed a significant difference in PSQI scores among groups (*p* = 0.02). This finding may be exploratory given the limited number of studies per subgroup and the risk of ecological bias when using study-level mean age. This finding may be due to the context dependencies of microbiota-sleep interactions, especially those associated with age, diet, and lifestyle (39). A recent study put forward an age-stratified perspective, highlighting that psychological stress in younger adults (aged 18–45) and immune changes in older adults (over 45 years old) may interact with age-related microbiome shifts to differentially influence sleep (40). While research links the gut microbiota to sleep quality and suggests potential benefits of probiotics, the mechanisms remain to be fully understood.

Subgroup analyses comparing intervention durations (4 weeks vs. >4 weeks) revealed no statistically significant differences between the two groups. The majority of the included studies (*n* = 12) did not assess whether sleep quality improvements were sustained beyond the intervention period. Of the two studies that included follow-up measurements, one study reported a significant between-group difference 3 weeks post-supplementation (17), while another found no such difference at the same time point (23). To better understand the durability of these improvements, future trials should incorporate follow-up assessments to evaluate their long-term benefits.

### 4.3 Additional findings

Regarding secondary outcomes, ISI scores were significantly reduced in the probiotic group compared to the control group, whereas no significant changes were observed on ESS scores or serum cortisol levels. These findings align with those of Gil-Hernández et al. (41), who also reported that probiotics failed to lower cortisol. The benefits of probiotics for sleep may be specific to perceived sleep quality and insomnia symptoms, rather than daytime sleepiness or physiological stress markers. Regarding other objective metrics, probiotic interventions prolonged sleep duration, enhanced deep sleep quality (increased *δ*-power and reduced N3 awakenings/latency), and decreased WASO.

### 4.4 Limitation

By incorporating the most recent trials published in 2024 and 2025, our research provides an up-to-date synthesis of the effects of probiotic interventions on sleep quality. Despite efforts to contact the authors by e-mail, certain studies were still excluded because the papers were inaccessible or the data were missing. Consequently, although a comprehensive literature search was conducted, the findings may not fully capture the true relationship owing to the absence of accessible studies. Given the relatively small number of included studies, the statistical power of the asymmetry tests is limited, and thus, this finding should be interpreted with caution.

Although several trials reported objective sleep measurements, methodological heterogeneity in outcome assessment precluded a formal meta-analysis. Standardizing the objective sleep measurement protocols across future studies would enhance the feasibility of evidence synthesis.

## 5 Conclusion

This meta-analysis found that probiotic supplementation is associated with a modest but statistically significant improvement in sleep quality among adults, regardless of baseline sleep status (ranging from healthy individuals to those with suboptimal sleep), as evidenced by the reductions in PSQI and ISI scores. However, probiotics did not significantly impact measures of daytime sleepiness (as assessed by ESS) or serum cortisol levels. These results suggest that the benefits of probiotics for sleep may be specific to perceived sleep quality and insomnia symptoms.

## Data Availability

The original contributions presented in the study are included in the article/Supplementary material, further inquiries can be directed to the corresponding authors.
