# Supplementary figures and images for: Association between probiotic intervention and sleep quality in the general adult population: a systematic review and meta-analysis
Source: Front Nutr. 2026 Mar 25;13:1795450. doi: 10.3389/fnut.2026.1795450 (PMC13057277; doi:10.3389/fnut.2026.1795450)

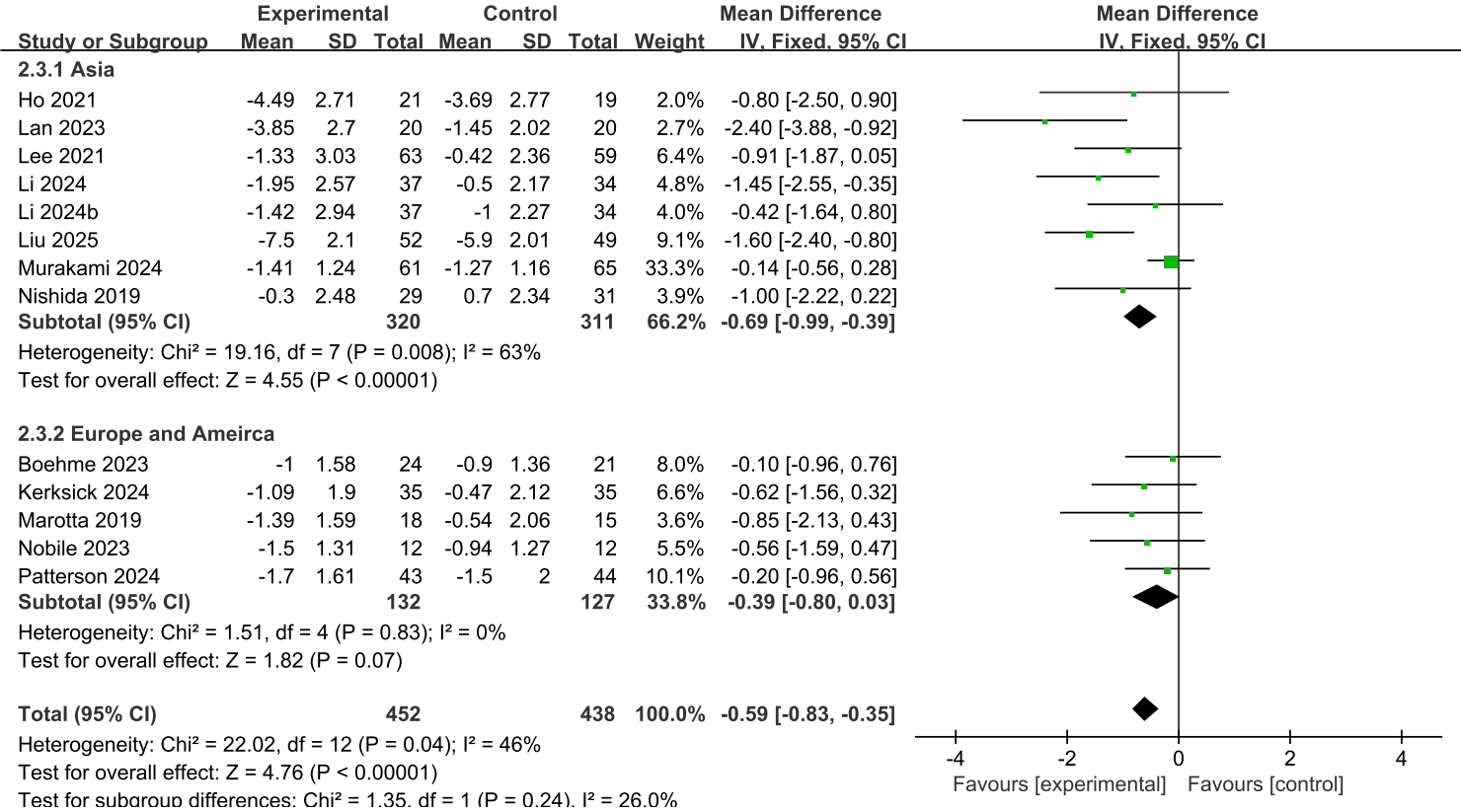

Supplement: Supplementary file 1 [file Data_Sheet_1.zip › supplement/Fig S1 Subgroup analysis of PSQI by source of participants.pdf]

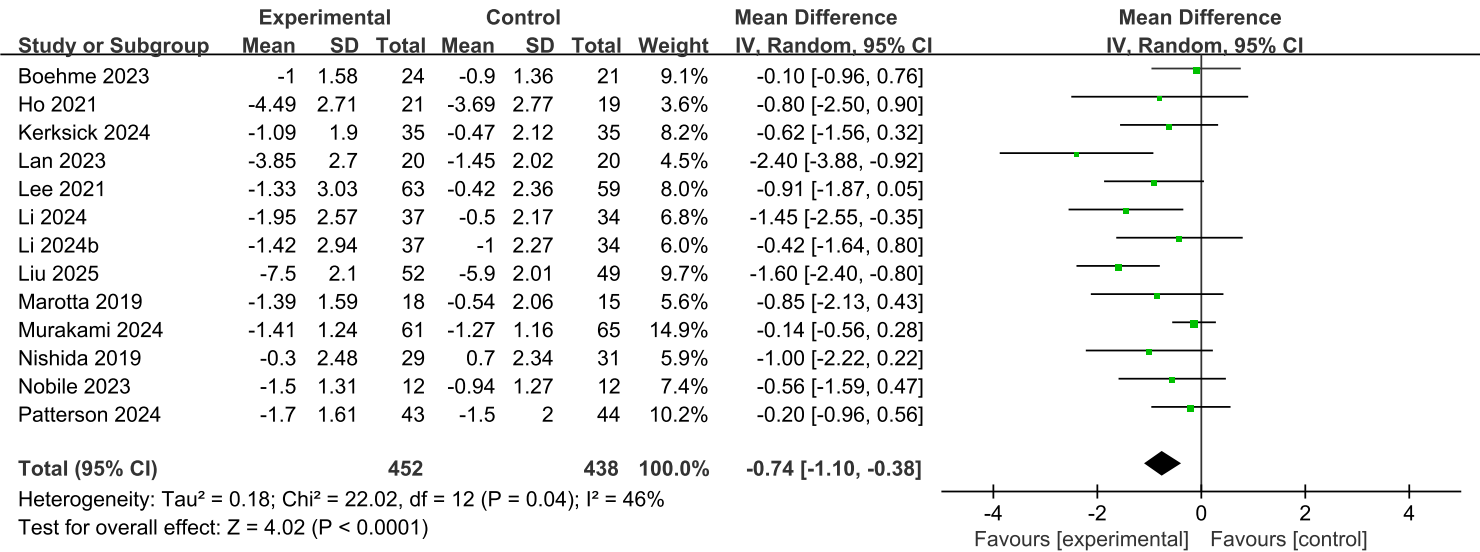

Supplement: Supplementary file 1 [file Data_Sheet_1.zip › supplement/Fig S10 Forest plot for PSQI based on a random-effects meta-analysis.pdf]

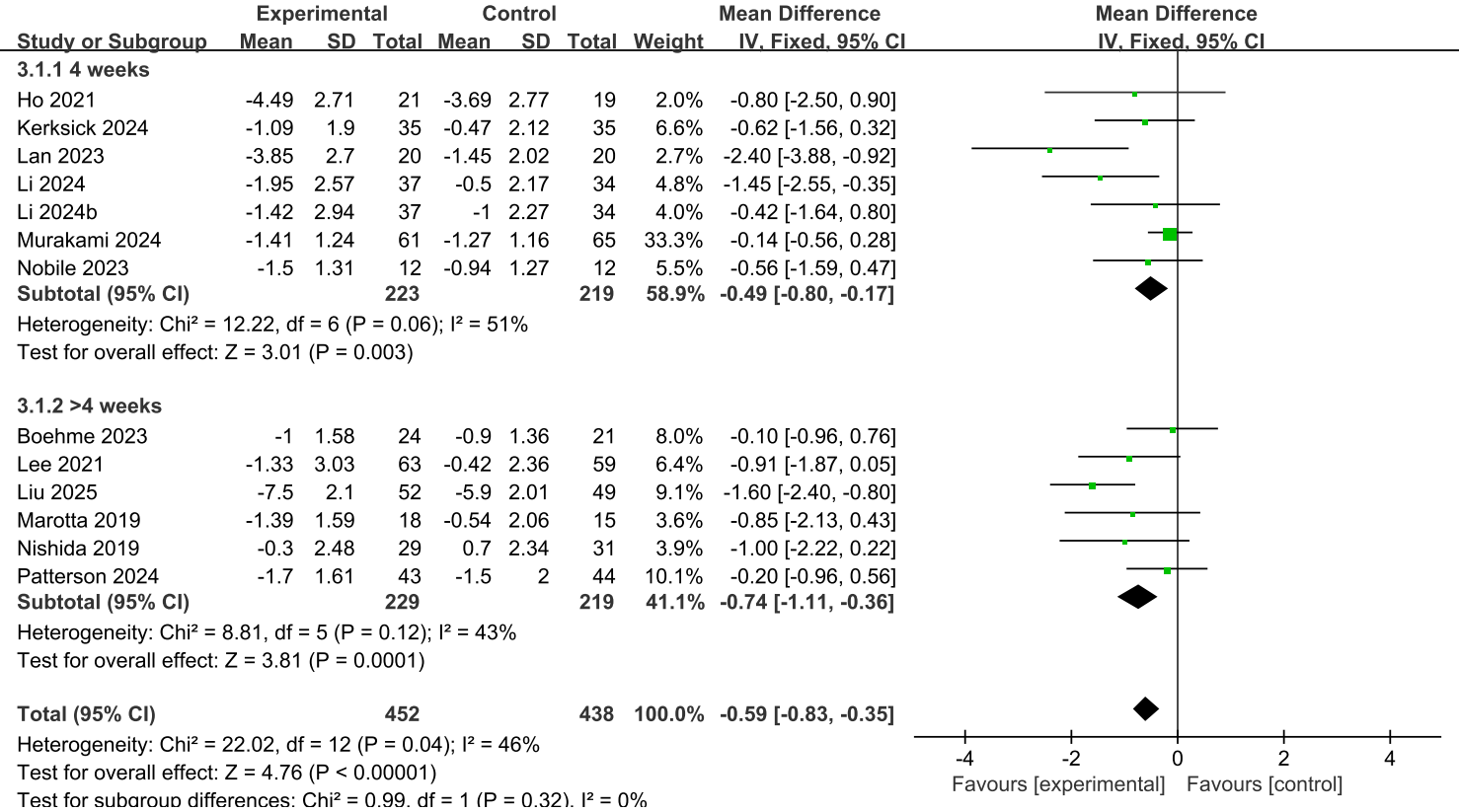

Supplement: Supplementary file 1 [file Data_Sheet_1.zip › supplement/Fig S2 Subgroup analysis of PSQI by intervention duration.pdf]

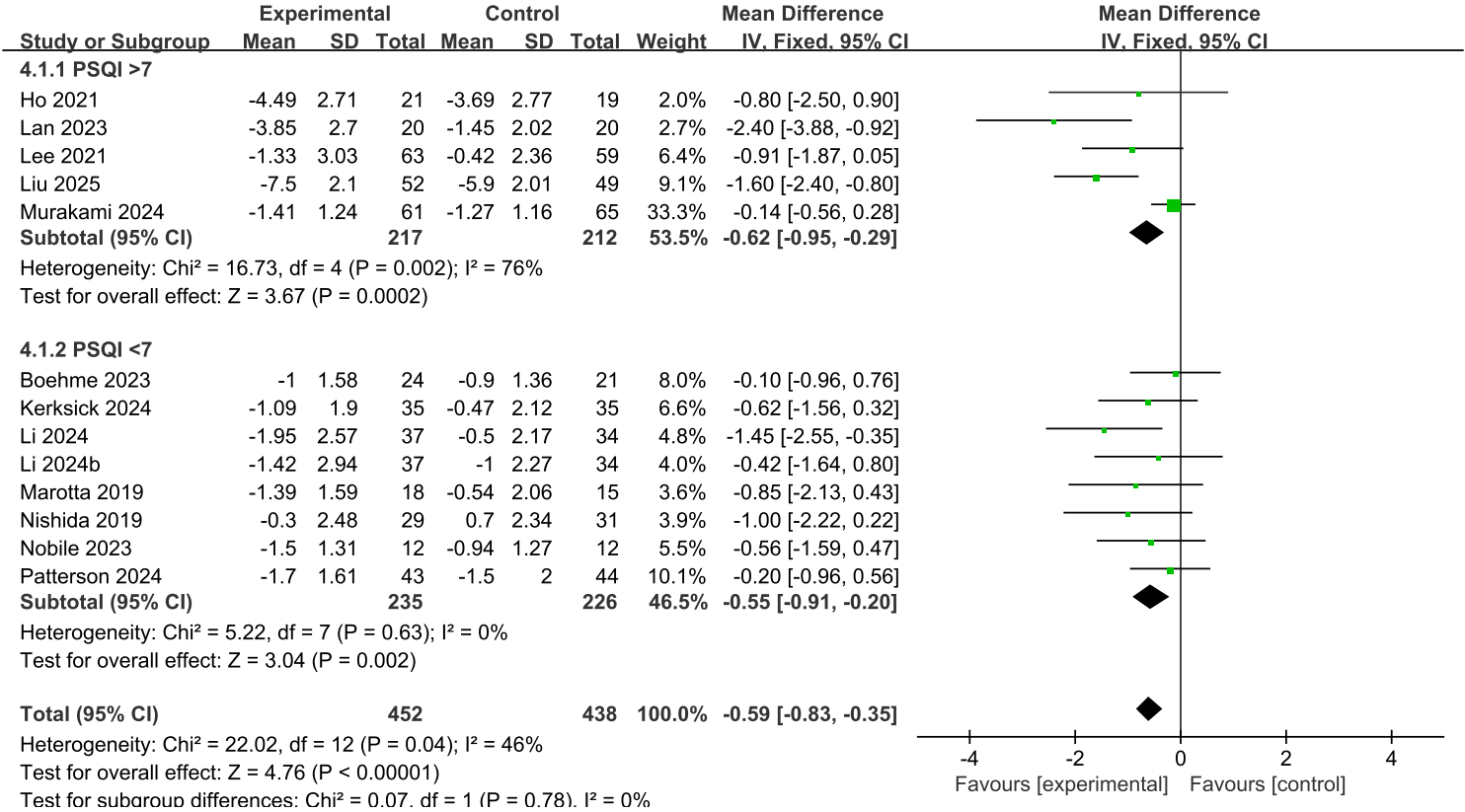

Supplement: Supplementary file 1 [file Data_Sheet_1.zip › supplement/Fig S3 Subgroup analysis of PSQI by baseline insomnia severity.pdf]

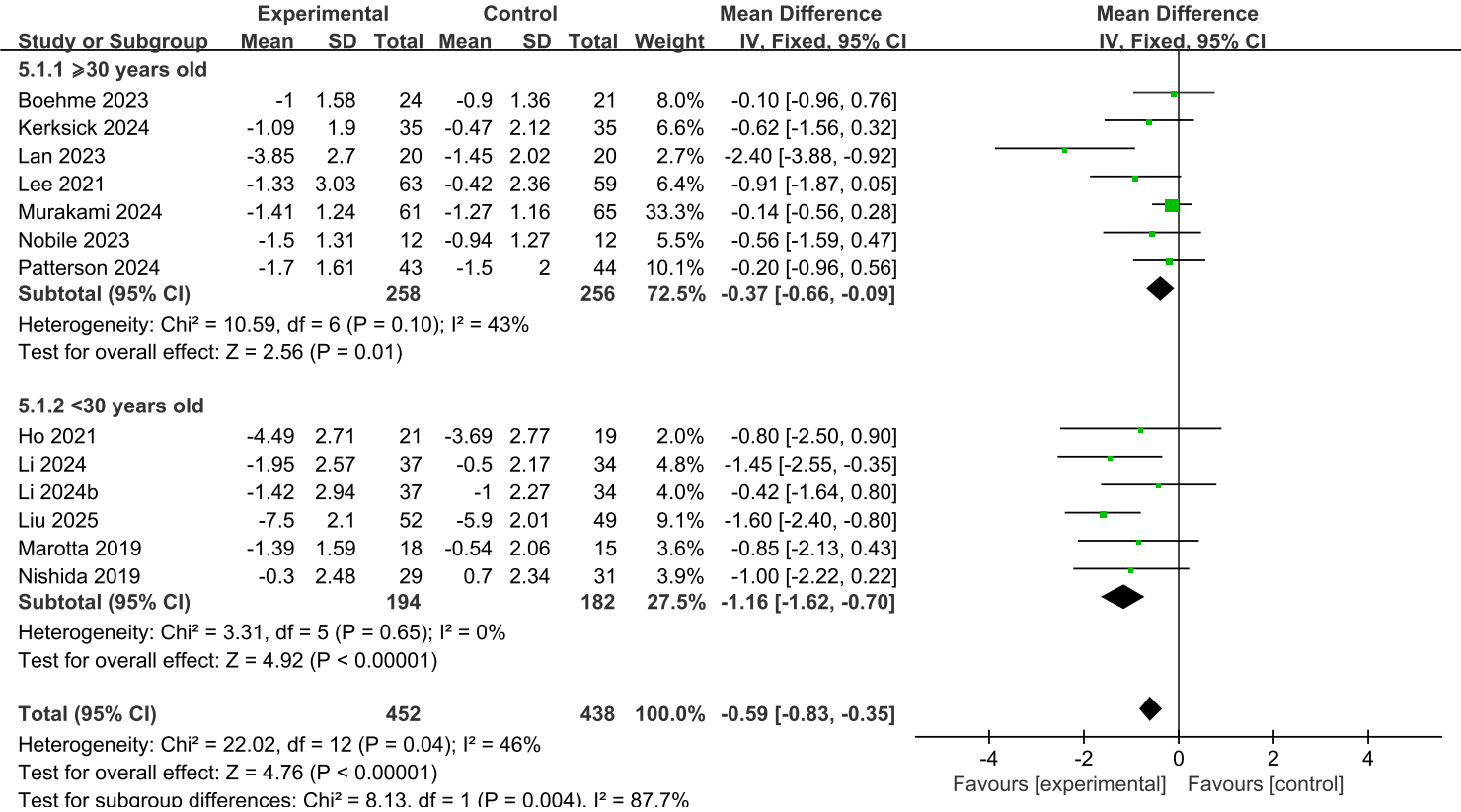

Supplement: Supplementary file 1 [file Data_Sheet_1.zip › supplement/Fig S4 Subgroup analysis of PSQI by mean age.pdf]

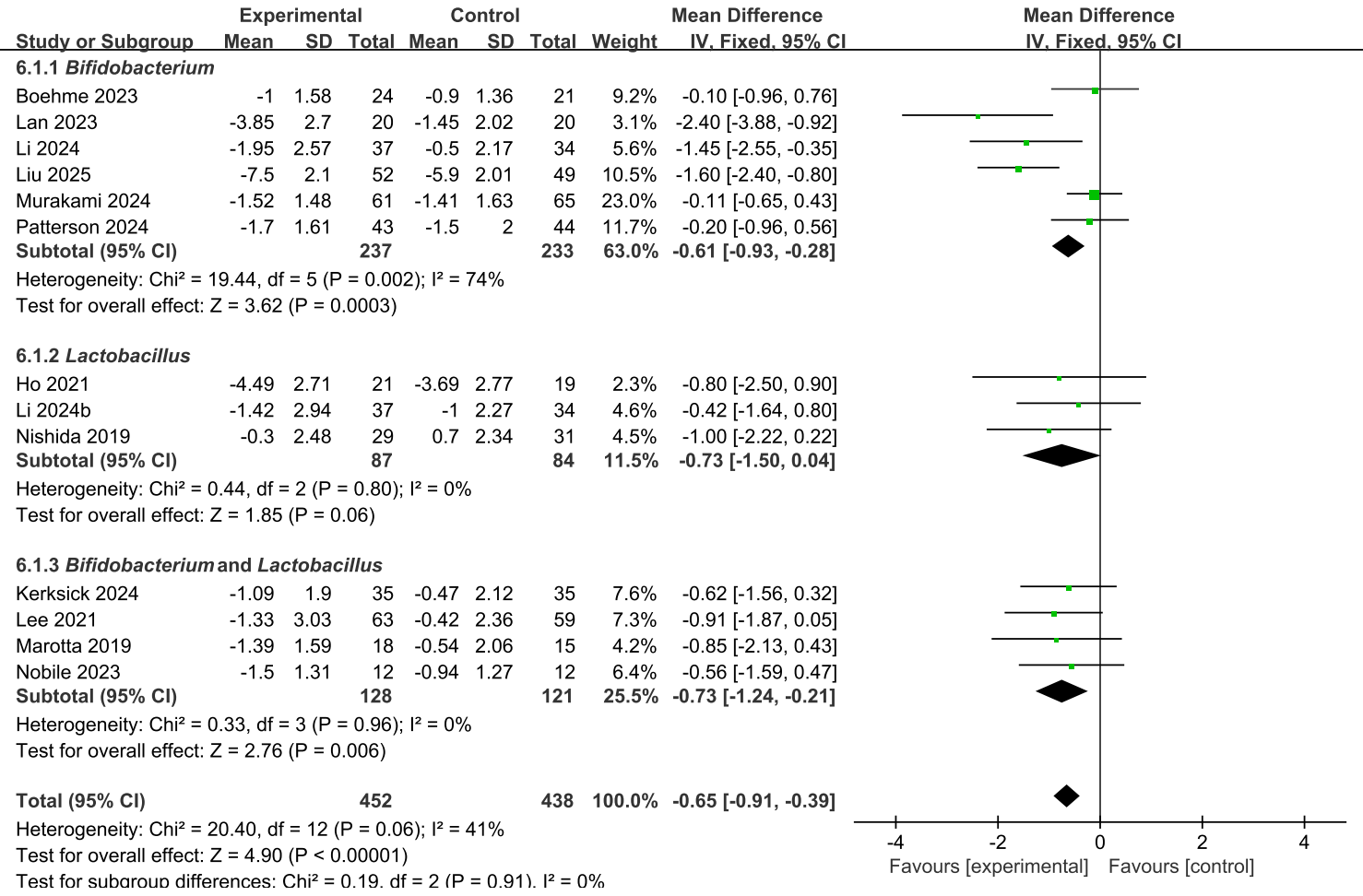

Supplement: Supplementary file 1 [file Data_Sheet_1.zip › supplement/Fig S5 Subgroup analysis of PSQI by genus.pdf]

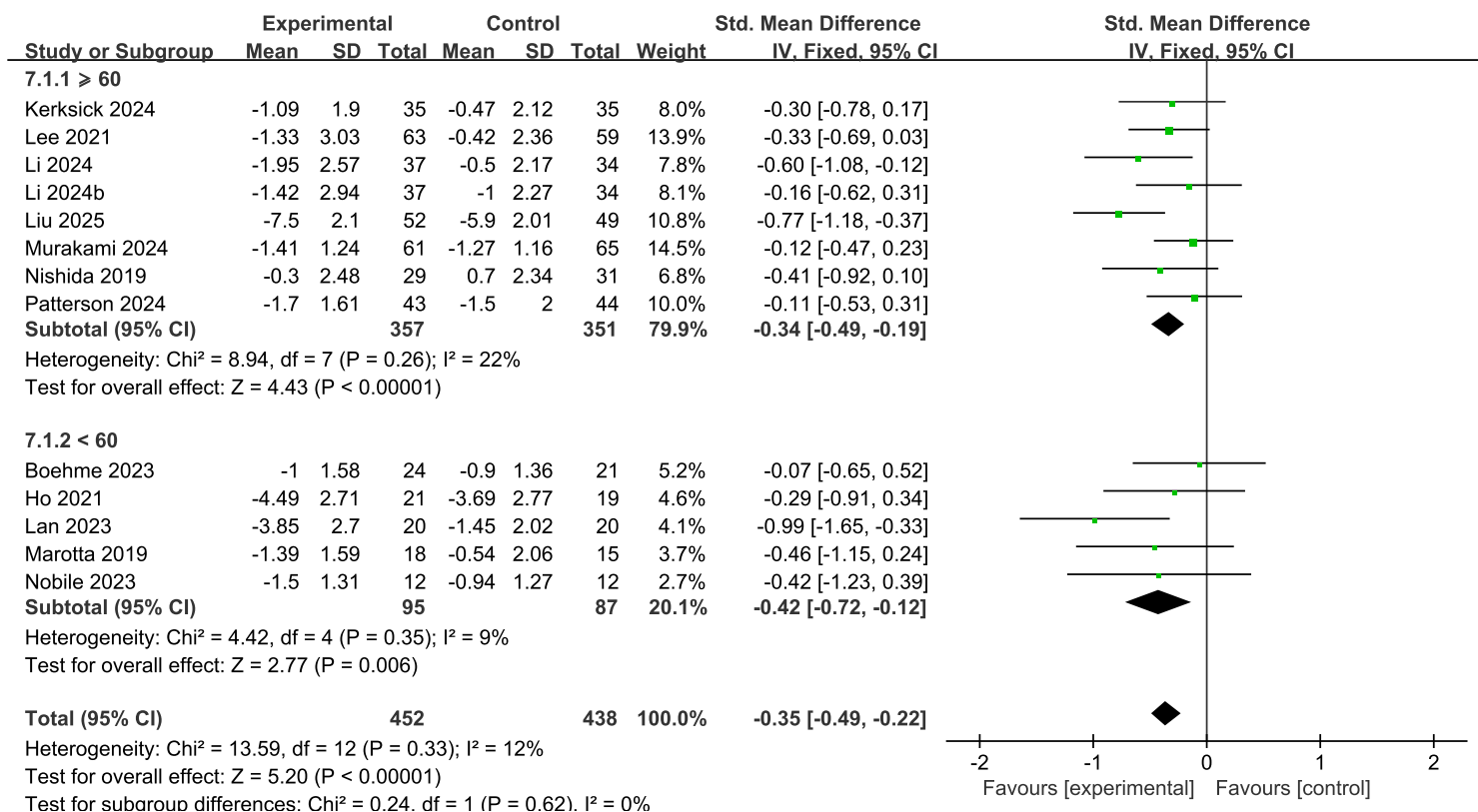

Supplement: Supplementary file 1 [file Data_Sheet_1.zip › supplement/Fig S6 Subgroup analysis of PSQI by sample size.pdf]

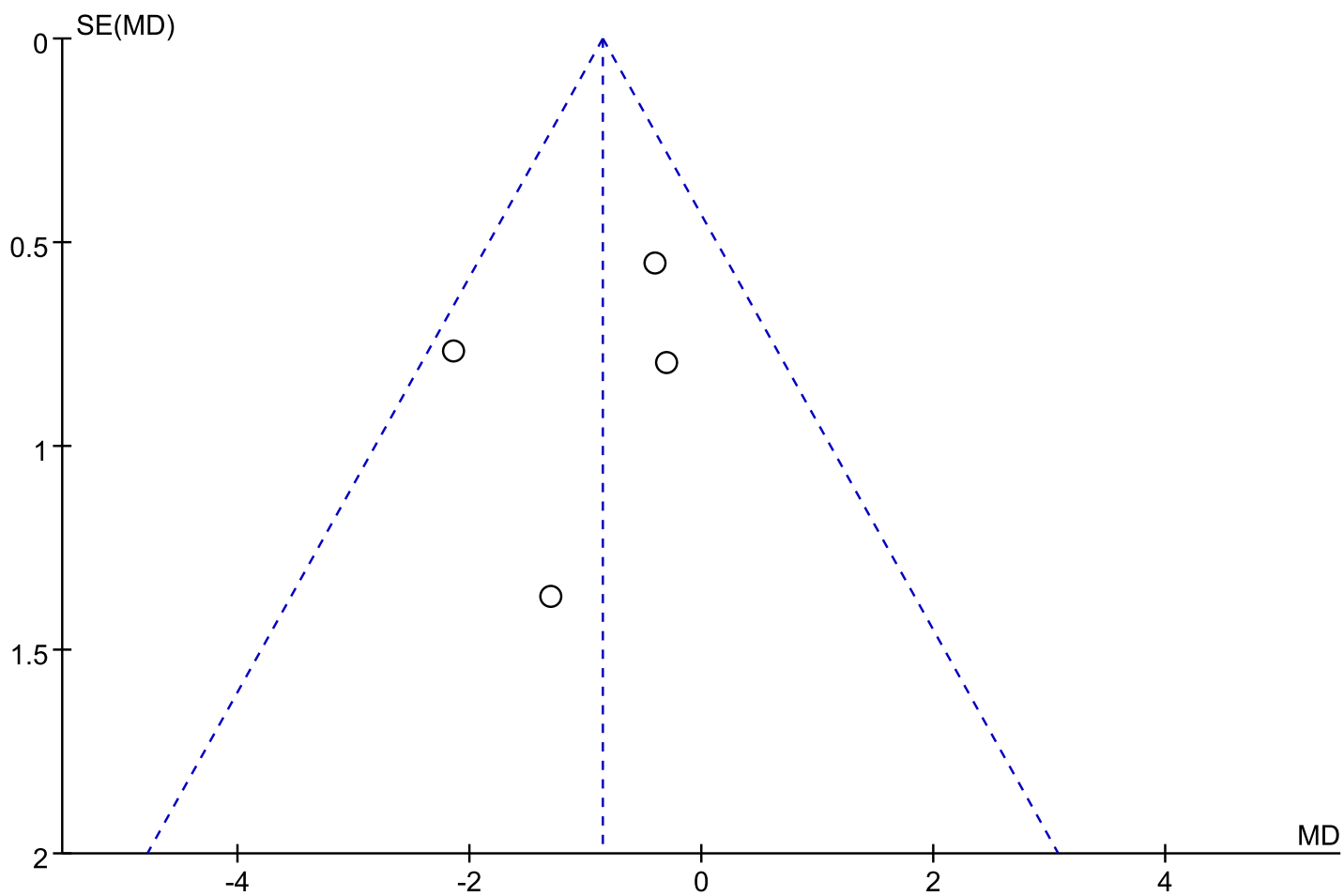

Supplement: Supplementary file 1 [file Data_Sheet_1.zip › supplement/Fig S7 Funnel plot of ISI.pdf]

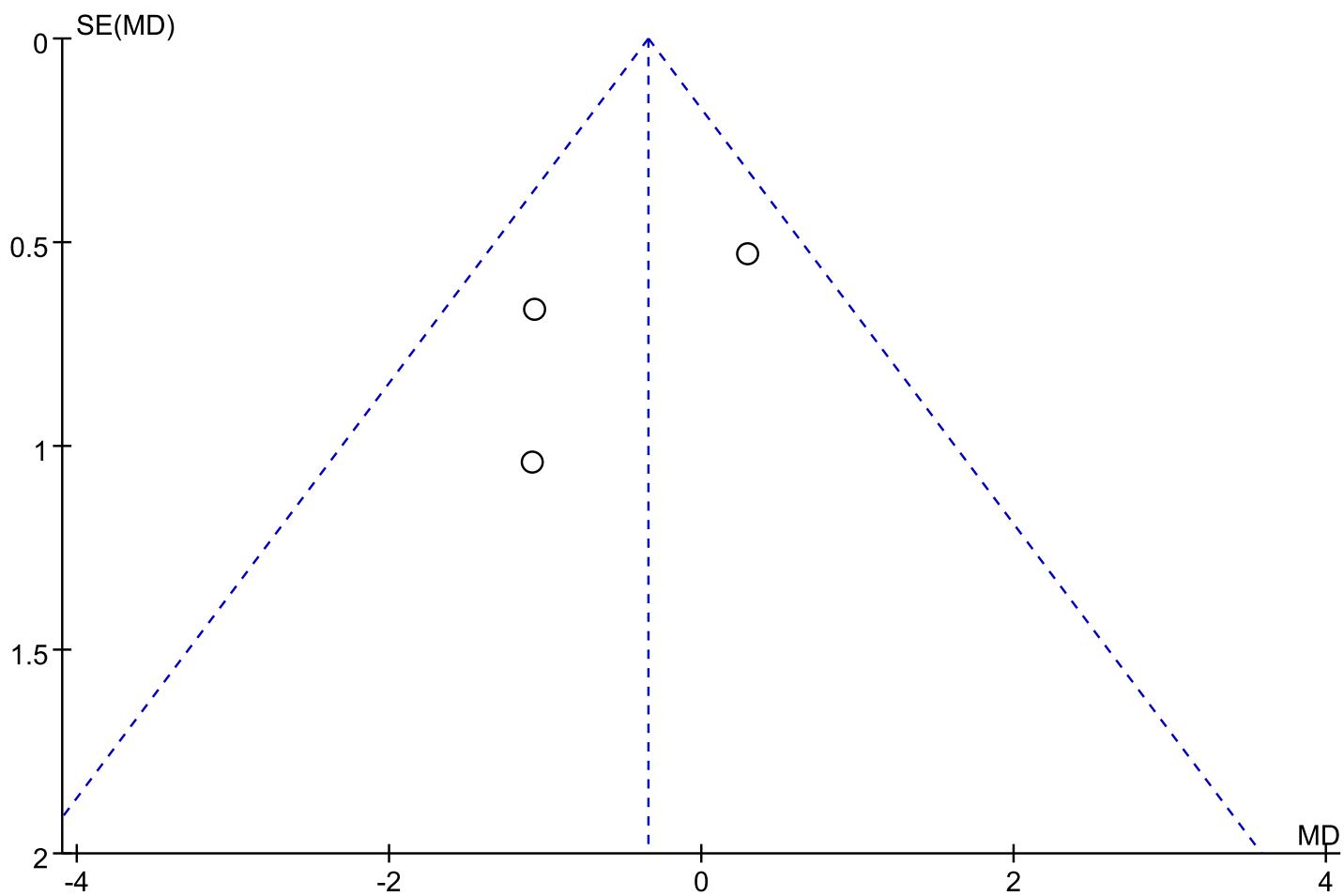

Supplement: Supplementary file 1 [file Data_Sheet_1.zip › supplement/Fig S8 Funnel plot of ESS.pdf]

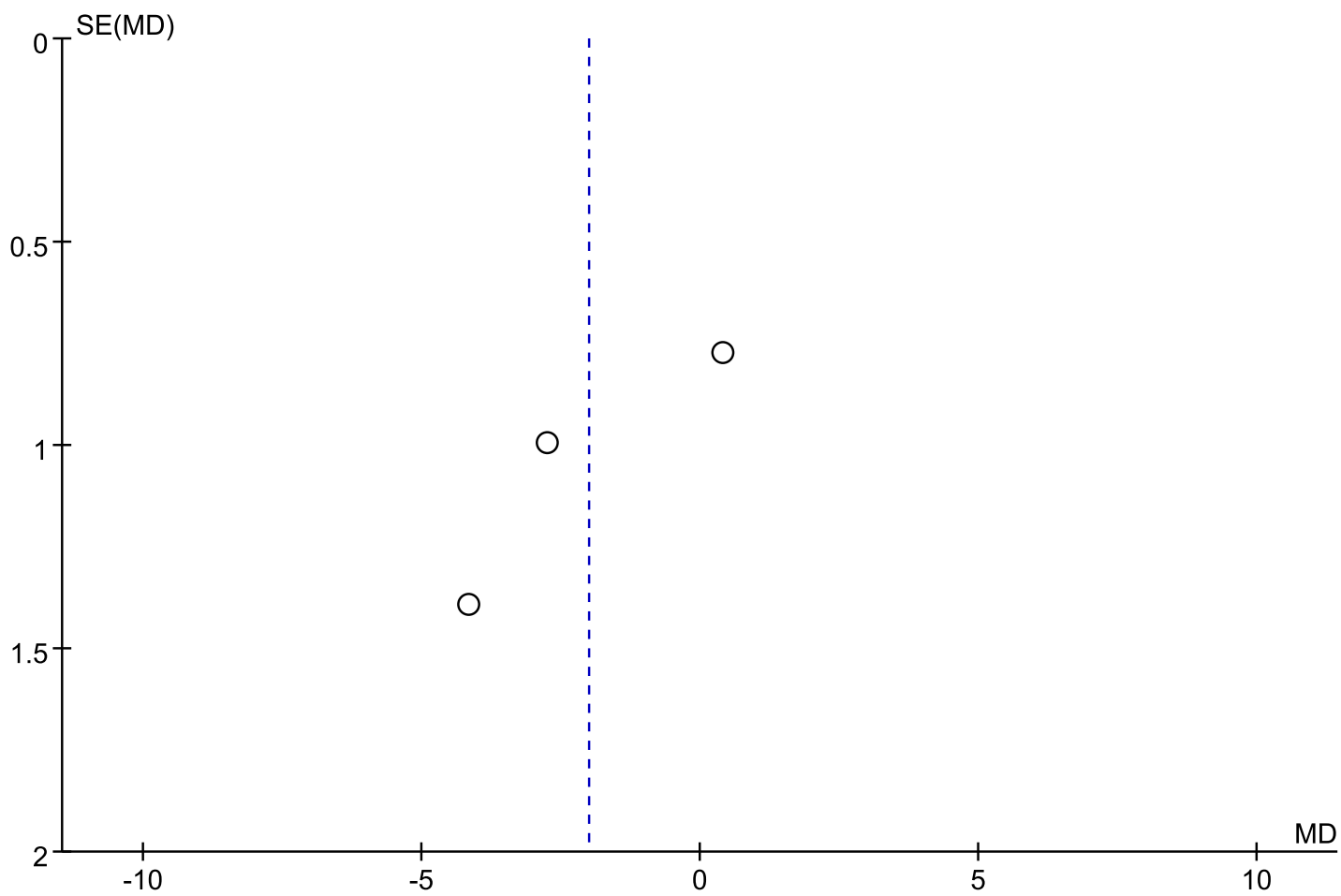

Supplement: Supplementary file 1 [file Data_Sheet_1.zip › supplement/Fig S9 Funnel plot of cor.pdf]
